# Adsorption Behavior of Magnetic Carbon-Supported Metal Nickel for the Efficient Dye Removal from Water

**DOI:** 10.3390/ijerph19031682

**Published:** 2022-02-01

**Authors:** Beifeng Lv, Jingjing Xu, Haibo Kang, Pengfei Liang, Wei Wang, Feifei Tao

**Affiliations:** 1 School of Civil Engineering, Shaoxing University, Shaoxing 312000, China; 20020852047@usx.edu.cn (B.L.); wellswang@usx.edu.cn (W.W.); 2 School of Chemistry and Chemical Engineering, Shaoxing University, Shaoxing 312000, China; xuliuri888@163.com (J.X.); pf13909425848@163.com (P.L.); 3 School of Civil Engineering, College of Transportation Engineering, Nanjing Tech University, Nanjing 210009, China; nobody@njtech.edu.cn

**Keywords:** template method, solvothermal method, carbon-supported metal nickel, adsorption performance

## Abstract

Magnetic carbon-supported metal nickel has been successfully synthesized by solvothermal method and heat treatment for highly effective adsorption of various reactive dyes. Structure characterization and composition analysis demonstrated that the metal nickel nanoparticles with the size of 1–2 nm were embedded into the pore of carbon spheres. It is helpful to prevent the agglomeration and falling off of metal nickel nanoparticles on the surface of carbon spheres. The adsorption performance of the carbon-supported metal nickel nanospheres for reactive brilliant yellow R-4GLN was studied by changing the pH value and initial concentration of the solution, adsorption time, adsorption temperature, and the amount of adsorbent. The carbon-supported metal nickel showed fast and efficient adsorption activity. After 5 min of adsorption, the removal efficiency of 10 mL 25 mg·mL^−1^ reactive brilliant yellow R-4GLN was close to 100%. The carbon-supported metal nickel composite was reused 20 times, and the removal efficiency of dye remained above 98%. It also showed good adsorption performance on various reactive dyes with wide universality, which has a certain adsorption effect on most dyes with a high utilization value in wastewater treatment.

## 1. Introduction

With the rapid growth of population and economy, environmental pollution has been an important problem affecting people’s healthy life. Water sources are becoming increasingly polluted due to the excessive discharge of colored wastewater from textile, paper, rubber, and other industries [1,2]. Therefore, efficient removal of colored wastewater is very important to alleviate environmental pollution [3,4,5]. Various technologies, such as ion-exchange [6], ozonation [7], chemical precipitation [8], electrochemical treatment [9], membrane separation [10], photocatalysis [11], and adsorption [12], have been developed to purify wastewater. Among then, adsorption has been considered economical and reliable due to its feasibility, easy operation, low cost, recyclability, and high efficiency [12,13,14].

The composite composed of carbon materials and magnetic metal has been proven to be used as excellent adsorbent to purify wastewater [15], due to the advantages of large specific surface area, easy regulation of size and morphology, stable performance, and easy recycling. Carbon materials from various carbon sources have been widely applied as adsorbents for the removal of inorganic and organic pollutions from water [16,17,18]. However, the separation and recovery of carbon materials is difficult, which limits its application. So as a new type of magnetic nanomaterial, the carbon-supported metal nickel composite also exhibits the potential application as adsorbent in wastewater treatment [19].

There are many preparation methods of metal nanoparticles, and the preparation of nano nickel powder mainly adopting the steam-condensation method, chemical vapor deposition method, precipitation method, sol-gel method, ionic liquid method, and solid-phase redox method, etc. [20]. Wu et al. [21] successfully synthesized spherical pure metal nickel nanoparticles by chemical reduction of nickel chloride with hydrazine at room temperature without any protective agent or inert gas, and the obtained nickel nanoparticles could be separated in solid state and stabilized in the atmosphere for several months. Chou et al. [22] prepared nickel nanoparticles by chemical reduction method under a controlled pH environment and also characterized the product. Meshkani et al. [23] prepared a series of nickel catalysts with different contents of supported mesoporous spherical SiO_2_ by sol-gel method, and the experimental results showed that the samples prepared with 55% nickel performed the best in the temperature and stability tests. Ádám et al. [24] used ultrasound-assisted reduction method to prepare nickel nanoparticles and carried out physical and chemical characterization. The experimental results showed that the ultrasonic prepared nickel nanoparticles had good catalytic activity. Liu Guang et al. [25] for the first time synthesized a graphene hybrid modified ultrafine nickel nanoparticle through in-situ chemical meteorological deposition to improve the wettability between metal and carbon nanophases. Sagadevan et al. [26] prepared nickel ferrite nanoparticles by co-precipitation method and characterized them. Ragupathi et al. [27] synthesized nickel aluminate nanoparticles by microwave combustion method (MCM) and found that nickel aluminate nanoparticles prepared by MCM had higher surface area and lower grain size than that prepared by conventional combustion method. Guillén-Bonilla et al. [28] synthesized nickel formic acid-resistant nanoparticles by microwave-assisted wet chemical method, which is a very economical and effective way to obtain the size of nanoparticles. Aghari et al. [29] synthesized nickel magnetic mirror nanoparticles by a convenient chemical method and studied their antibacterial effect on *S. aureus* and *E. coli* bacteria. Krebsz et al. [30] synthesized recently carbon microsphere-supported metallic nickel nanoparticles by carbonizing a polystyrene-based cation exchange resin loaded with nickel ions. Nanoparticles showed high catalytic activity in nitrophenol reduction.

Due to the characteristics of low redox potential, active properties, magnetic properties, and easy agglomeration of nickel, it is still difficult to prepare nano-nickel powder with small particle size, uniform dispersion, and good performance [31]. The template synthesis method allows the generated nanoparticles to be assembled in a certain way, so as to obtain supramolecular nanomaterials with specific morphologies [32]. There are relatively few reports on the preparation of nano metal powders by this method, especially for carbon-supported metal powder with magnetic properties. The physical and chemical properties of nanomaterials largely depend on their size and shape. Due to the special physical and chemical properties of nickel, nickel and its compounds have been widely used in chemical, aviation, military, and construction engineering fields [33,34,35], but there are few reports on water pollution treatment in the field of environmental geotechnical engineering. Therefore, the preparation of carbon-supported metal nickel and its application in water treatment have become the focus of this thesis.

In this paper, a simple template method combined with annealing treatment is proposed to synthesize magnetic spherical carbon-supported nickel. UV-vis spectrometer is used to study the adsorption performance of carbon-supported metal nickel for reactive dyes, by changing the pH value and initial concentration of the solution, adsorption temperature, adsorption time, and the amount of adsorbent to explore the best conditions for the adsorption of reactive brilliant yellow R-4GLN by carbon-supported metal nickel and its universality. Here the introduction of a large number of metal nickel with 1–2 nm in the surface of carbon spheres improves the specific surface area, exhibits the paramagnetic properties for the separation and recovery of the adsorbent, and enhances the adsorption performance.

## 2. Experimental Section

### 2.1. Preparation of Carbon Spheres

Carbon spheres with size of about 200 nm were prepared following literature methods with some modifications [36]. 2.0 g of anhydrous glucose was dispersed in 20 mL of distilled water, and then stirred until the glucose was completely dissolved. The above solution was transferred to a stainless-steel autoclave and kept at 180 °C for 8 h. After the reaction, the autoclave was cooling to room temperature naturally, and the black powder was centrifuged and washed with a large amount of distilled water. Then it was put in an oven to dry at 80 °C for about 10 h to obtain the carbon sphere template for preparing the carbon-supported nickel composite.

### 2.2. Preparation of Carbon-Supported Nickel Composite

Carbon-supported metal nickel composite was prepared on basis of previously reported work with some modifications [36]. 0.06 g of NiCl_2_·6H_2_O and 0.10 g of the prepared carbon spheres were dispersed in the mixed solution of 10 mL distilled water and 10 mL absolute ethanol. After the ultrasonic treatment for 10 min, the above suspension was added by 2.52 g of C_2_H_2_O_4_·2H_2_O, which was placed in ultrasound for another 2 h and stirred for 10 h at the room temperature. The obtained suspension was centrifuged and washed with distilled water five times, and then it was placed in an oven to dry at 80 °C overnight. The product was put into the tubular furnace and kept at 350 °C for 2 h for annealing treatment under the protection of N_2_ to obtain the carbon-supported nickel composite.

### 2.3. Characterization Methods

The morphology of the samples was characterized by scanning electron microscopy (SEM, JSM-6360LV, from Japan, JEOL) and high-resolution transmission electron microscope (HRTEM, JEM-2100F, from Japan, JEOL). The energy dispersive X-ray spectroscopy (EDS) was used to determine the elements on an energy spectrum (X-act, from UK, Oxford). The crystal structures of the products were characterized by X-ray diffractometer (XRD, XRD-6000, from Netherlands, Empyrean, Cu K α λ = 0.15406 nm). The content of metal nickel in the composite was determined by inductively coupled plasma atomic emission spectrometry (ICP-AES, from USA, Leeman) The Brunauer–Emmett–Teller (BET) surface areas and porosities were measured by N_2_ adsorption–desorption isotherms on an Empyrean apparatus from USA Micromeritics. Magnetic hysteresis curve was evaluated using a vibrating sample magnetometer (VSM attachment on PPMS Dynacool System, from USA, Quantum Design International) at room temperature.

### 2.4. Adsorption Experiments

To investigate the adsorption properties of the as-prepared samples, 10 mg of the powder was dispersed in 10 mL 25 mg·L^−1^ of reactive dye solution (reactive brilliant yellow R-4GLN, reactive dark blue B-2GLN or reactive red R-4BD) as the dye wastewater by the ultrasound treatment for at least 2 min. There was no equilibrium time spent before starting the adsorption tests. After each period of time, 5 mL of the suspension was taken out and centrifuged. To obtain the concentration of dye solution, the obtained supernatant was evaluated using a calibration curve plotted with UV-vis spectrometer (UV1800TC), and the absorbance at the maximum absorption wavelength of each dye was used to calculate the removal efficiency. To ensure the accuracy of the measurement, each experimental data is obtained by repeating three times, and the average value of the three data is used.

(1) $$Removal\%=\left(A_{0}-A\right)/A_{0}\times 100\%$$

where, *A*_0_ represents the absorbance of the reactive dye at the maximum absorption wavelength before adsorption, and *A* represents the absorbance of the reactive dye at the maximum absorption wavelength after adsorption.

The adsorption capacity and equilibrium constant are calculated as follows:

(2) $$q=\left(C_{0}-C_{e}\right)*V/m$$

(3) $$C_{e}/q=1/\left(K*q_{m}\right)+C_{e}/q_{m}$$

where, *q* represents the quantity of dye per mass of adsorbate at equilibrium (mg/g); *C*_0_ represents the initial concentration of the solution (mg/L); *C*_e_ represents the concentration of the dye solution at equilibrium (mg/L); V represents the volume of the adsorption solution (mL); *m* represents the amount of adsorbent (g); *q*_m_ represents the maximum Langmuir monolayer adsorption capacity (mg/g); and *K* represents the Langmuir constant (L/mg).

### 2.5. Cyclic Stability Measurement

To make clear the cyclic stability of the carbon-supported metal nickel composite for the adsorption of reactive brilliant yellow R-4GLN, 20 consecutive adsorption test cycles were conducted at the same experimental conditions. After each cycle, the recovered composite was washed with a large amount of distilled water, dried at 80 °C, and heat treated at 350 °C for 2 h for the desorption of dye molecules.

## 3. Results and Discussion

### 3.1. Morphology and Structure Characterization

**Figure 1:**
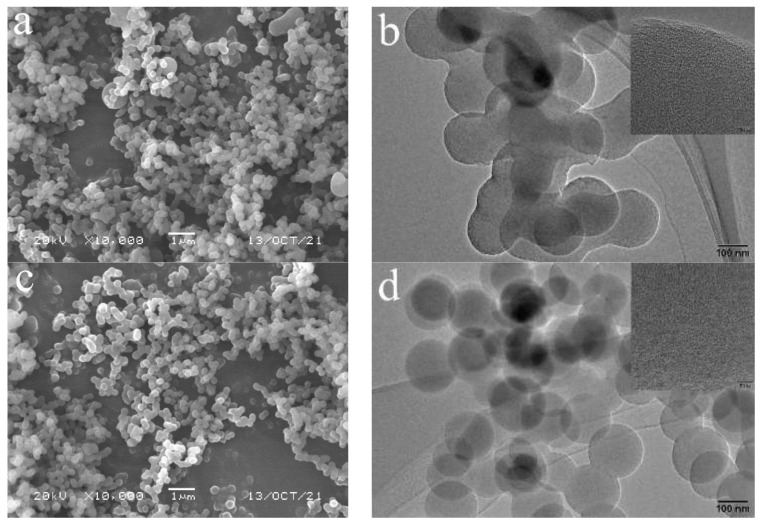
SEM (**a**,**c**) and HRTEM (**b**,**d**) images of carbon spheres (**a**,**b**) and carbon-supported metal nickel (**c**,**d**).

**Figure 2:**
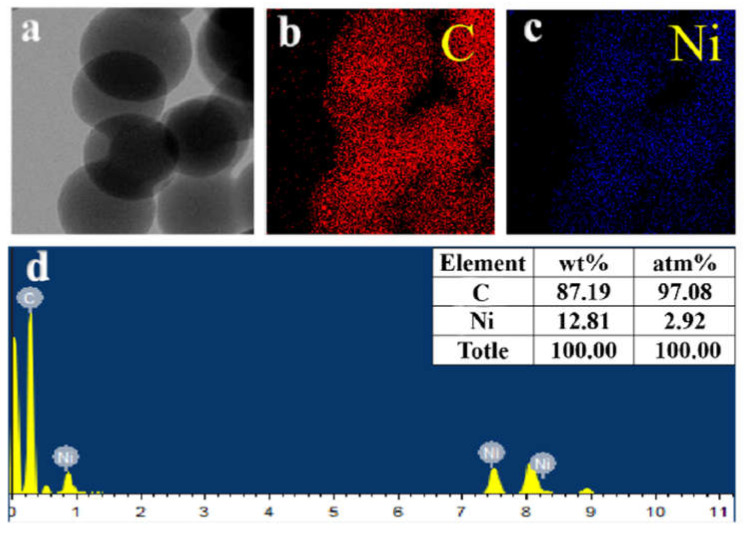
[M7] HRTEM image (**a**), the corresponding element mapping of C (**b**) and Ni (**c**) elements, EDS pattern (**d**) and elemental contents (inset) of carbon-supported metal Ni.

Figure 1 shows the SEM and TEM images of carbon spheres and carbon-supported metal nickel. As shown in Figure 1a, there are a large number of spherical particles with the uniform diameter of 200–300 nm, which can be proved by HRTEM image (Figure 1b). It is further found that the spheres intersect with each other, resulting in poor dispersion. From the further enlarge HRTEM image (inset of Figure 1b), no lattice fringe can be observed, indicating that the product is weakly crystalline or amorphous carbon. When the metal nickel is loaded on the carbon spheres, the obtained product exhibits the morphology and size similar to carbon spheres (Figure 1c). Based on the further observation from the HRTEM image (Figure 1d), it is found that the spheres are complete and don’t intersect with each other, indicating the good dispersion. From the enlarge HRTEM image (inset of Figure 1d), many black dots are scattered on the surface of sphere independently of each other, indicating that metal nickel nanodots with the diameter of 1–2 nm embed in the pore of the spheres, which is further proved by the element mapping analysis of C and Ni elements over the carbon-supported metal nickel (Figure 2a–c). The Ni metal displays the element mapping similar to C element, indicating that the metal nickel nanodots are evenly distributed on the surface of carbon spheres. Moreover, based on the investigation of the surface composition by EDS (Figure 2d), it is found that the obtained sample consists of C and Ni elements, further confirming the HRTEM results. And the content of Ni elements is 12.81 wt%, which is similar to 12.90 wt% calculated by the addition during the reaction and 12.72 wt% from ICP-AES. The unique structure of the carbon-supported metal nickel can effectively protect metal nickel from oxidation.

**Figure 3:**
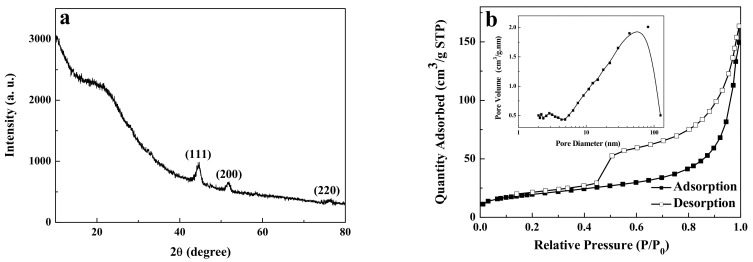
XRD pattern (**a**), and nitrogen adsorption–desorption isotherm and pore size distribution (inset) of carbon-supported metal nickel (**b**).

**Figure 4:**
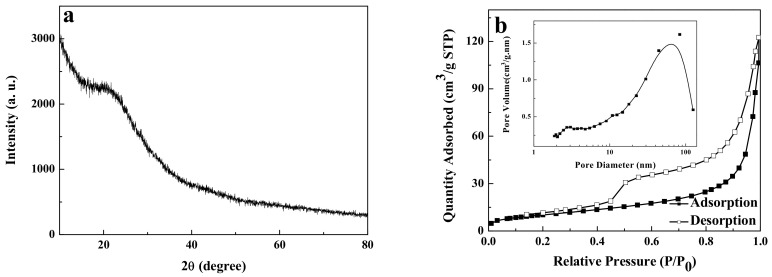
XRD pattern (**a**), and nitrogen adsorption–desorption isotherm (**b**) and pore size distribution (inset) of carbon spheres.

Figure 3a shows the XRD pattern of carbon-supported metal nickel, in which the diffraction peaks appear near 44.5°, 51.8°, and 76.4°. Compared with the standard pattern of face-centered cubic Ni (JCPDS No. 04-0850) [37], these diffraction peaks correspond to (111), (200), and (220) crystal planes of metal Ni, indicating that metal nickel is face-centered cubic structure. According to the Scherrer formula [36], the average particle size of metal nickel nanocrystals is deduced to be about 2.4 nm, which is in good agreement with the results of HRTEM. Moreover, there is a protrusion between 15–30°, which can be attributed to the presence of amorphous carbon phase [19,36]. Compared with the XRD curves of carbon spheres (Figure 4a), this protrusion is attributed to carbon spheres. Due that the carbon spheres are composed of the amorphous carbon, no obvious peak of carbon is found in the XRD curves. In addition, no other diffraction peaks are found in the figure, indicating that the nickel element is completely reduced to metal nickel. This implies that the presence of metal nickel in the carbon-supported metal Ni composite is due to the reducing atmosphere formed in the preparation process. Furthermore, the existence of amorphous carbon in the composite plays a protective role and further prevents the oxidation of metal nickel [37]. The as-prepared carbon-supported metal nickel composite has strong magnetic properties and is helpful for the separation and recovery of adsorbents in water treatment applications [38,39,40].

Based on the morphology observation, the porous nature and specific surface area of the samples should be investigated by the N_2_ adsorption–desorption isotherms and pore size distribution. The carbon-supported metal nickel with 70.35 m^2^·g^−1^ shows a higher specific surface area than the carbon spheres (37.46 m^2^·g^−1^), which may be due to the introduction of metal nickel nanodots with small size. As shown in Figure 3b and Figure 4b, both the carbon-supported metal nickel composite and carbon spheres show type IV isotherms and the hysteresis loop at the *P*/*P*_0_ value of 0.20–1, indicating the existence of mesoporous structure [41,42]. The porous features from the surface cavities of carbon spheres and the interleaving spheres are further verified by the pore size distribution from the BJH model [43]. For the carbon-supported metal nickel, the pore diameter of the carbon-supported metal nickel is measured to be about 2.82 nm and 56.24 nm (inset of Figure 3b). The mesopores smaller than 50 nm may be produced by the heat treatment, and the large pores may be due to the accumulation of nanospheres [44]. The carbon spheres have the similar N_2_ adsorption–desorption isotherms to the carbon-supported metal nickel, but have the larger pores at 2.99 nm and 65.16 nm, leading to the lower specific surface area. Moreover, a large number of independent and complete spheres are produced, which is helpful to improve the specific surface area. The above result is in good agreement with the SEM and HRTEM results.

**Figure 5:**
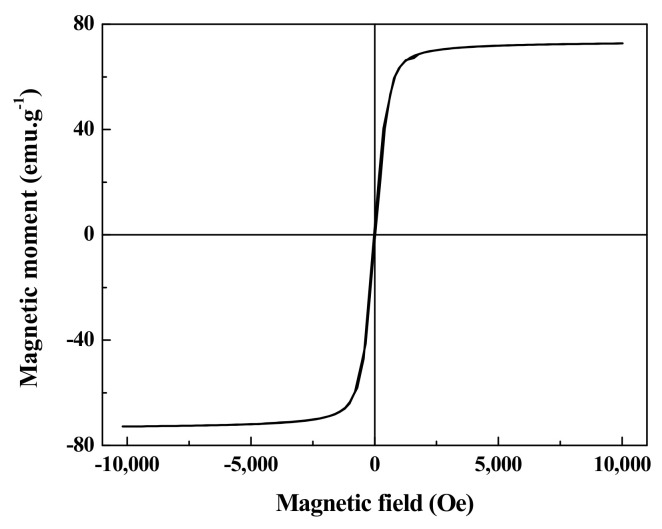
The magnetic hysteresis loops of the carbon-supported metal nickel.

The magnetic measurement of the carbon-supported metal nickel is shown in Figure 5. There is no obvious hysteresis for the sample, indicating the paramagnetic behavior at room temperature [45]. The saturation magnetization is measured to be 72.68 emu·g^−1^, which can ensure the separation and recovery of the adsorbent in practical application [46]. Compared with the traditional filtration and centrifugation, the magnetic carbon-supported metal nickel is easily separated and recovered by applying an external magnetic field, exhibiting promising potential in large scale industrial applications.

### 3.2. Adsorption Performance

#### 3.2.1. Effect of pH Value

The pH value plays an important role in investigating the adsorption performance of the carbon-supported metal nickel composite [45]. The reactive brilliant yellow R-4GLN solution with an initial concentration of 25 mg/L was prepared with distilled water. The pH values of the initial solution were adjusted to 1, 1.45, 1.62, 2, 3, 4, 5, 6, 7, and 8 by concentrated hydrochloric acid, 0.1 mol/L of hydrochloric acid, or 0.1 mol/L of NaOH solution, and oscillated for 2.5 h at a constant temperature of 308 K.

**Figure 6:**
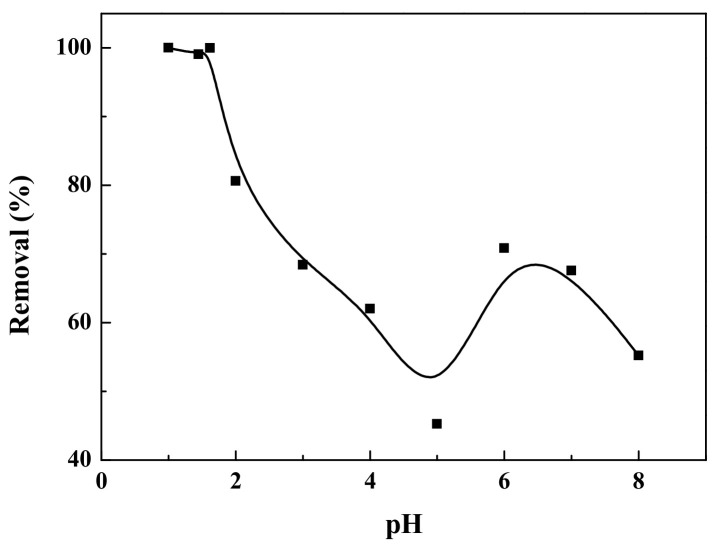
The adsorption effect of carbon-supported metal nickel on reactive brilliant yellow R-4GLN under different pH values.

Figure 6 shows the effect of pH value on the removal efficiency of reactive brilliant yellow R-4GLN solution over carbon-supported metal nickel composite. It can be seen from the figure that the pH value of the solution has a significant effect on adsorption performance. The lower the pH value, the better the adsorption performance. When pH value is lower than 2, the removal efficiency is close to 100%. With the increasing pH value, the removal efficiency first decreases and then increases. When pH = 5, the removal efficiency is 45.26%, which reaches the lowest value. Then, as the pH value increases, the removal efficiency increases slightly, but it does not exceed 75%. In an acidic environment, the composite is protonated and the surface of the adsorbent is positively charged, leading to the maximum adsorption of anionic dyes [47,48]. So, the decrease of pH value means the enlarged positive potential of the composite, which results in the increasing adsorption capacity for anionic dye [47,48]. In summary, the adsorbent has a better adsorption effect when the pH value is small. When pH = 1.62, it reaches adsorption saturation. Considering the adsorption performance and economic cost, the pH value of the solution was set at 1.62 in this experiment.

#### 3.2.2. Effect of Contact Time

**Figure 7:**
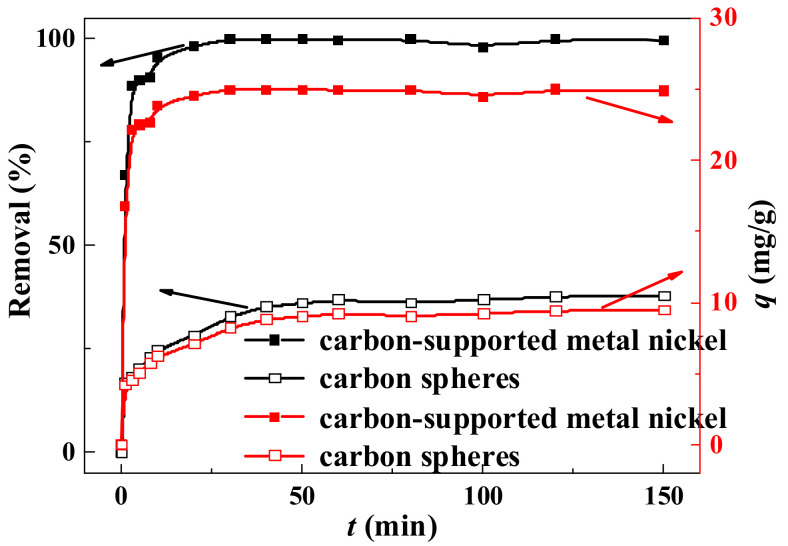
Effect of contact time on the removal of reactive brilliant yellow R-4GLN over carbon spheres and carbon-supported metal nickel.

The adsorption performance was influenced by variable contact times. As shown in Figure 7, at the beginning of adsorption, the initial rate of adsorption removal increases rapidly with the extension of contact time. For carbon-supported metal nickel, more than 95% of reactive brilliant yellow R-4GLN is removed within the contact time of 5 min, and nearly 100% is removed within the contact time of 20 min, displaying the quick and effective adsorption. However, for carbon spheres, only 35% of reactive brilliant yellow R-4GLN is removed under the adsorption for 40 min. When the adsorption reaches equilibrium, only 38% is removed. This is proved by the adsorption capacity curve at Figure 7. Therefore, compared with carbon spheres, the carbon-supported metal nickel exhibits the efficient and rapid adsorption, which reveals that a quick monolayer formation occurred on the external surface of the composite [48]. And the excellent adsorption performance for the magnetic composite may be due to the large specific surface area caused by the unique morphology and structure.

#### 3.2.3. Effect of Initial Dye Concentration

**Figure 8:**
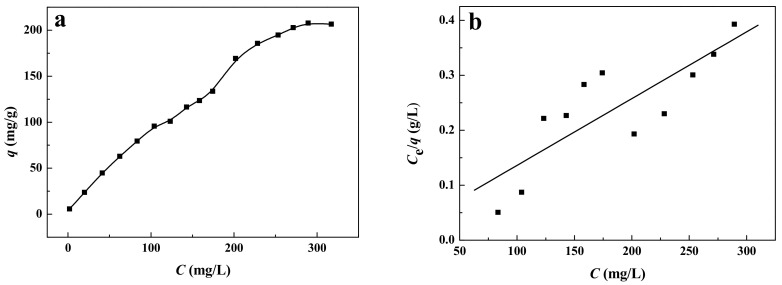
(**a**) Effect of the initial dye concentration on adsorption performance and (**b**) *C*_e_/*q*-*C* curve.

The effect of initial dye concentration on the adsorption performance was investigated in a range from 1 to 325 mg/L. To ensure the accuracy of initial dye concentration, 1 g/L of reactive brilliant yellow R-4GLN solution was prepared with distilled water, and then diluted to different concentrations. The pH value of the solution was adjusted to 1.62 with concentrated hydrochloric acid and oscillated at a constant temperature of 308 K for 5 h. Figure 8a shows the effect of the initial concentration of the dye solution on adsorption performance. It can be seen from the figure that the dye removal displayed a change trend of rapid increase at first and then gradually becoming stable with the increase of the initial concentration. At the low concentration, the dye molecules are easily adsorbed on the available surface of the adsorbent [48]. However, at high dye concentration, the surface active sites of the adsorbent are saturated, which causes the adsorption to become slow [48]. When the initial concentration is 290 mg/L, the adsorption capacity at equilibrium is 207.78 mg/g.

The adsorption properties of the as-prepared magnetic carbon-supported metal Ni were compared with previously reported adsorbents in Table 1. Various carbon-based adsorbents can effectively remove the organic pollutions from wastewater under their own experimental conditions. Compared with other carbon-based adsorbents, the as-prepared magnetic carbon-supported metal Ni composite exhibits the good adsorption property with the adsorption capacities of 207.78 mg/g for reactive brilliant yellow R-4GLN.

In order to achieve the effective and rapid adsorption, investigation of the adsorption kinetics is very necessary and important. The Langmuir model is fitted according to the adsorption isotherm, and the adsorption isotherm can be obtained from Figure 8b by the equation *C*_e_/*q* = 0.0012*C*_e_ + 0.0146 (*R*_2_ = 0.682), where *C*_e_ is the concentration of dye solution at equilibrium. From this equation we can calculate *q_e_* = 833 mg/g, *K*_L_ = 0.0822 L/mg, where *q*_e_ is the quantity of dye per mass of adsorbate at equilibrium (mg/g) and *K*_L_ (L/mg) is the Langmuir constant [48]. The basic characteristics of Langmuir model can be described by the infinite dispersion constant (*R*_L_ = (1 + *KC*_0_)^−1^) [57], where *R*_L_ is also known as equilibrium parameter, *C*_o_ is the initial concentration (mg/L), and *K* is the Langmuir constant related to the energy of adsorption (L/mg) [48]. The reliability of Langmuir equation is determined according to *R*_L_ value. When 0 < *R*_L_ < 1, it indicates that the obtained data basically conforms to Langmuir model. When *R*_L_ = 1, the obtained data conforms to Langmuir model, and the obtained Langmuir equation is reliable. When *R*_L_ = 0 or *R*_L_ > 1, it shows that the obtained data do not conform to Langmuir model, and the obtained Langmuir equation is not reliable [58]. According to the calculated *R*_L_ data as shown in Table 2, the experimental data basically conforms to Langmuir model.

#### 3.2.4. Effect of Adsorption Temperature

**Figure 9:**
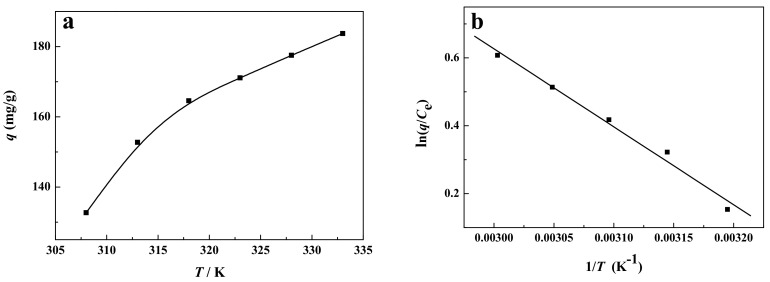
(**a**) Effect of adsorption temperature on removal of dye and (**b**) ln(*q*/*C*_e_) - 1/*T* curve.

Figure 9a displays the effect of the adsorption temperature on the removal of the reactive brilliant yellow R-4GLN solution with an initial concentration of about 25 mg/L at the pH value of 1.62. It can be seen from Figure 9a that the removal efficiency of the reactive brilliant yellow R-4GLN over carbon-supported metal nickel increases with the increasing adsorption temperature. This may be attributed to the enhanced mobility of dye that is provided by the sufficient energy to interact with the available active sites of adsorbent molecule [48]. Therefore, the adsorption properties are enhanced at the higher temperature. By the ln(*q*/*C*_e_)~1/*T* curve in Figure 9b, the fitting equation can be obtained as follows.

(4) $$y=-2297.2x+7.51\;\left(R2=0.9861\right)$$

According to the line slope −∆*H*/*R*, the enthalpy changes ∆*H* = 19.098 kJ/mol in the range of 308–333 K can be calculated, indicating that the adsorption process is an endothermic process. Therefore, the increase of the temperature is very beneficial to the adsorption. Due to the enthalpy change value at the range of 0.418~62.7 kJ/mol, the adsorption performance of carbon-supported metal nickel on reactive brilliant yellow R-4GLN can be interpreted mainly by physical adsorption. On the other hand, higher temperature can enhance the desorption of dye due to violent molecular motion, leading to the decrease of adsorption capacity. Thus, the temperature is kept at 308 K.

#### 3.2.5. Effect of Adsorbent Dosage

**Figure 10:**
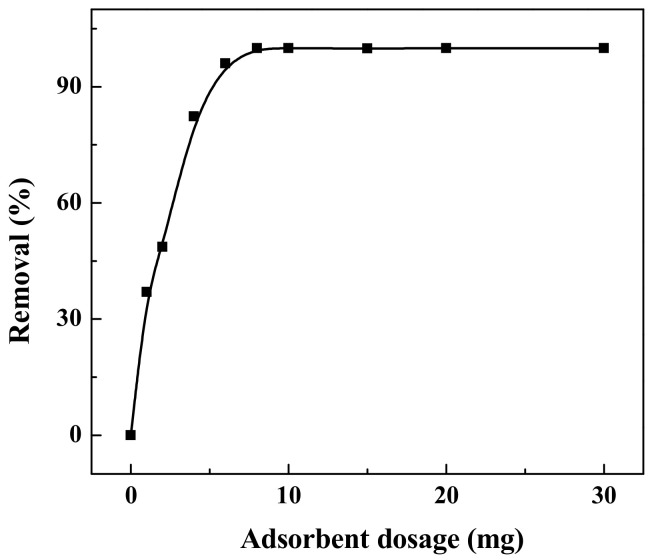
Effect of the adsorbent dosage on the adsorption performance.

The effect of adsorbent mass is usually used to investigate the solid adsorbent’s capacity at a given initial concentration of adsorbate in a solution. Figure 10 shows the effect of the amount of adsorbent on the removal of reactive brilliant yellow R-4GLN solution with 25 mg/L and pH = 1.62. It can be seen from the figure that when the amount of adsorbent is less than 8 mg, the removal efficiency increases with the increasing amount of adsorbent. When the amount is 1 mg, only 36.97% of dye is removed. With the adsorbent amount of 4 mg, about 82.30% of dye can be removed. And when the amount is more than 6 mg, the removal of dye is close to 100%. This phenomenon occurs mainly because with the increase of adsorbent mass, the amount of adsorbed active brilliant yellow R-4GLN increases accordingly. The amount of adsorbent dosage plays a crucial role in the dye removal process [48].

#### 3.2.6. Universality of Adsorbent

To investigate the universality of carbon-supported metal nickel composite, various dyes like reactive violet X-2R, reactive turquoise blue BF-BGN, reactive golden yellow B-4RFN, reactive dark blue B-2GW, reactive black P-BR and reactive red R-4BD with an initial concentration of about 25 mg/L were removed at a constant temperature of 308 K. UV-visible analysis of each dye solution before and after adsorption was carried out in the same way to calculate the removal efficiency of various dyes.

**Figure 11:**
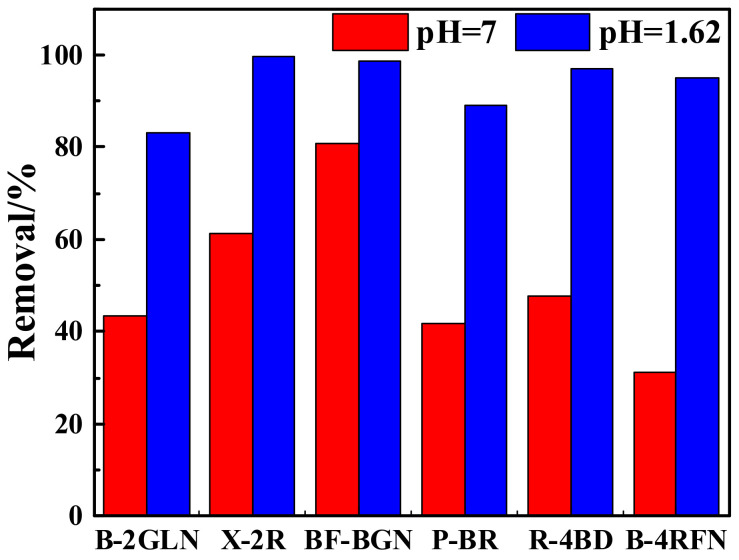
The adsorption performance of carbon-supported metal nickel on various dyes under neutral conditions and pH = 1.62.

It can be seen from Figure 11 that carbon-supported metal nickel exhibits the good adsorption performance on various dyes, and the relative data are shown in Table 3. When pH = 1.62, the removal efficiency of the reactive violet X-2R solution, reactive turquoise blue BF-BGN solution, reactive golden yellow B-4RFN solution, and reactive red R-4BD solution can reach more than 95%, and that of reactive black P-BR solution can reach 89%, and that of reactive dark blue B-2GW solution is also 83%. Under neutral condition, the removal efficiency of reactive turquoise blue BF-BGN solution can also reach 80%, while that of other dyes is relatively low. It can be seen that carbon-supported metal nickel has universal applicability for the adsorption of various reactive dyes, and the adsorption performance under acidic conditions is better than that under neutral condition. The research results show that carbon-supported metal nickel has potential application value in wastewater treatment.

#### 3.2.7. Cyclic Stability

**Figure 12:**
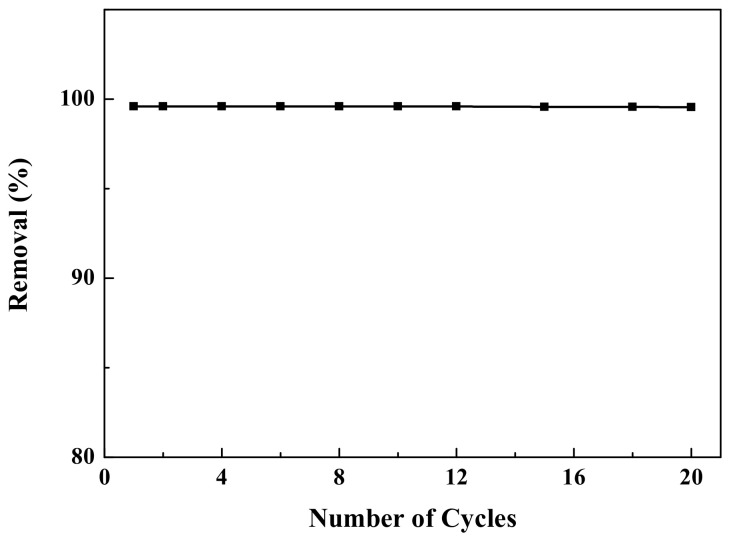
Effect of number of cycles on the removal efficiency of reactive brilliant yellow R-4GLN solution over carbon-supported metal nickel.

**Figure 13:**
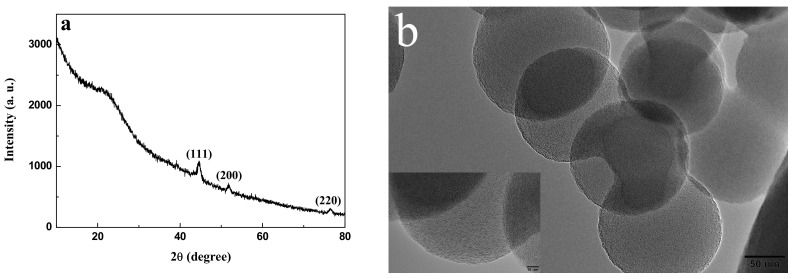
XRD pattern (**a**) and HTEM image (**b**) of the used carbon-supported metal nickel after 20 cycles. The inserted figure in (**b**) is a partially enlarged view of the sample.

To evaluate the reproducibility and stability of the carbon-supported metal nickel, the adsorption test of 20 times were carried out in Figure 12. It is found that after 20 cycles, the removal efficiency of reactive brilliant yellow R-4GLN solution is still close to 100%, indicating the good stability of carbon-supported metal nickel. Based on the XRD and HRTEM images of the carbon-supported metal nickel after 20 cycles (Figure 13), it is found that the used carbon-supported metal nickel remains the similar crystal structure and morphology to the unused sample. Moreover, the average particle size of metal nickel nanocrystals is about 2.5 nm and the content of metal nickel in the used composite is about 12.62 wt%, in good agreement with the fresh sample, indicating the good stability and reusability.

## 4. Conclusions

In this paper, the carbon-supported metal nickel magnetic nanomaterials were synthesized on a large scale, and the adsorption performance on various reactive dyes has been studied. The preparation is simple and easy to operate. The magnetic adsorbent is easy to be separated and recovered from the solution under an external magnetic field. Compared with carbon spheres, the carbon-supported metal nickel displays good adsorption performance for various reactive dyes in wastewater. When pH = 1.62, the removal efficiency is close to 100%, and the adsorption rapidly reaches saturation within 5 min. In the pH range of 2–8, as the pH value increases, the removal efficiency decreases slightly, and both exceeds 45%, indicating that carbon-supported metal nickel can be used as adsorbent in a variety of different pH systems. The adsorption performance of carbon-supported metal nickel nanomaterials conforms to Langmuir model with good universality. In this paper, a carbon-based adsorbent with a simple synthesis method and low cost was constructed, and its high adsorption performance indicates its potential application in dye wastewater treatment.

## Figures and Tables

**Table 1:** Carbon-based adsorbents for the removal of organic pollutions.

Carbon-Based Adsorbents	Organic Pollutions	*q*_m_ (mg/g)	Reference
Magnetic carbon-supported metal Ni	Reactive brilliant yellow R-4GLN	207.78	This work
Magnetite/carbon adsorbents from apple	Methyl blue Congo red Rhodamine B	97.85 ± 18.14 500.55 ± 174.96 254.77 ± 144.84	[49]
Magnetite/carbon adsorbents from banana	Methyl blue Congo red Rhodamine B	135.72 ± 13.34 433.83 ± 297.38 270.71 ± 83.26	[49]
Magnetite/carbon adsorbents from orange peels	Methyl blue Congo red Rhodamine B	183.05 ± 58.51 337.98 ± 94.61 196.14 ± 53.51	[49]
Layered double hydroxide-carbon dot composite	Methyl blue	185	[50]
Activated carbon based on synthetic and agricultural wastes	Methyl orange	38.83	[51]
Carboxylic acid-terminated carbon nanoflakes	Methylene blue Crystal violet Rhodamine B	~148 ~132 ~118	[52]
Activated carbon-based multicarboxyl adsorbent	Rhodamine 6G	122.55	[53]
Carbon nanotube incorporated eucalyptus derived activated carbon-based adsorbent	Methylene blue Eosin yellow	49.61 49.15	[54]
Sugarcane bagasse carbon-based composite	Methylene blue Methyl violet	261 125	[55]
Candle soot coated polyurethane foam	Rhodamine B	15.066	[56]

**Table 2:** Effect of the initial dye concentration *C*_0_ on the infinite dispersion constant R_L._.

*C*_0_ (mg·L^−1^**)**	2	20	41	62	83	104	123	143	158	174	202	228	253	271	289	317
*R*_L_	0.87	0.38	0.23	0.16	0.13	0.10	0.09	0.08	0.07	0.07	0.06	0.05	0.05	0.04	0.04	0.04

**Table 3:** Removal efficiency of various dyes over carbon-supported metal nickel at pH = 7 and pH = 1.62.

Condition	Reactive Dark Blue B-2GLN	Reactive Violet X-2R	Reactive Turquoise Blue BF-BGN	Reactive Black P-BR	Reactive Red R-4BD	Reactive Golden Yellow B-4RFN
Removal efficiency at pH = 7 (%)	43.37	61.46	80.72	41.85	47.79	31.10
Removal efficiency at pH = 1.62 (%)	83.15	99.83	98.82	89.19	96.95	95.05

## Data Availability

The data presented in this study are available on request from the corresponding author.
